# Validation of measurement scale for technostress in Peruvian university students

**DOI:** 10.3389/fpsyg.2025.1503442

**Published:** 2025-03-18

**Authors:** Emilyn Verde-Avalos, Josué Edison Turpo-Chaparro, Joel Palomino-Ccasa, Gabriela Requena-Cabral, Segundo Salatiel Malca-Peralta

**Affiliations:** ^1^Escuela de Posgrado, Universidad Peruana Unión, Lima, Peru; ^2^Escuela de Psicología, Universidad Peruana Unión, Lima, Peru; ^3^Escuela de Educación, Universidad Peruana Unión, Lima, Peru

**Keywords:** technostress, academic stress, well-being, mental health, higher education, psychometrics

## Abstract

Stress has become one of the major health issues worldwide, and the mandatory integration of technologies into the educational environment has exacerbated this problem. This situation affects both teachers and students, who often lack the necessary knowledge to use these tools effectively, putting their mental health and emotional well-being at risk across various areas. Over the years, numerous studies have been conducted on technostress in both workplace and educational contexts, analyzing its causes and consequences, as well as psychometric evaluations of instruments related to technological adaptation. This study presents an instrumental design and cross-sectional approach, with 814 Peruvian university students voluntarily participating, consisting of both sexes (451 females; 55%) aged between 18 and 36 years (*M* = 25.2, SD = 5.12). The instrument used was the Technostress Questionnaire for University Students (TS4US), initially developed for Chinese university students, which underwent a cultural adaptation process, expert judgment, and pilot testing. The exploratory factor analysis (EFA) and confirmatory factor analysis (CFA) of the TS4US versions revealed that the 11-item version maintained the three original dimensions, showing an excellent fit with indices of CFI = 0.982, TLI = 0.975, RMSEA = 0.007, and SRMR = 0.02, along with high internal consistency, with Alpha and Omega coefficients greater than 0.86. These results confirm that the TS4US is a valid and reliable tool for measuring technostress in Peruvian university students.

## 1 Introduction

The concept of stress was introduced into the field of health in 1926 by Hans Selye, who defined it as a physiological response of the organism to situations perceived as threatening or challenging (Selye, 1993). This response can trigger physical and emotional alterations, as well as serious pathologies such as depression, which affect the quality of life (Mariotti, 2015). In the academic context, the term “academic stress” refers to the high pressure experienced by students, which is associated with mental health problems such as anxiety and sleep disorders, resulting from academic demands and performance expectations (Carreón-Gutiérrez et al., 2024).

Recent studies highlight the effects of academic stress on the well-being and health of university students (Barbayannis et al., 2022). High levels of stress can trigger physical symptoms, such as dermatitis, the intensity of which is positively correlated with the level of stress (Kiupel et al., 2023). Additionally, academic stress causes depression, which negatively affects students’ academic performance (Deng et al., 2022) and also results in significant emotional changes (Clabaugh et al., 2021). These adverse effects impact both mental health and academic productivity, affecting students’ overall well-being.

In this context, the advancement of Information and Communication Technologies (ICT) has profoundly transformed the process of socialization, where family, the educational system, and ICT have assumed primary roles (Collins and Halverson, 2018). The COVID-19 pandemic accelerated this transformation, driving the transition to virtual education, which created new sources of stress for both students and teachers (Toscano et al., 2024). This abrupt shift heightened students’ psychological vulnerability, increasing anxiety and stress due to the dynamics of online learning (Churampi-Cangala et al., 2024). For teachers, the adaptation was perceived as imposed, increasing concerns about the proper use of ICT and remote teaching (Wang and Li, 2019).

These events gave rise to the concept of “technostress,” referring to the stress generated by excessive or inappropriate use of ICT, which affects both academic and work settings (Bahamondes-Rosado et al., 2023). Technostress has been described as a syndrome that negatively impacts academic productivity, especially due to factors such as technological overload, the complexity of digital tools, and insecurity in their use (Ragu-Nathan et al., 2008). However, studies suggest that digital literacy, technical support, and active participation can mitigate its effects (Ioannou, 2023). Proper management of technostress can open growth opportunities, helping individuals improve their digital skills and adapt to technological changes more effectively.

The effects of technostress can be severe, manifesting as mental exhaustion, lower academic performance, and sleep disorders, also affecting physical health (Toscano et al., 2024). This syndrome significantly decreases academic engagement and negatively impacts satisfaction with the learning process (Wang et al., 2020). Three subtypes of technostress have been identified: techno-anxiety, characterized by heightened physiological arousal; techno-fatigue, which describes cognitive exhaustion after prolonged use of ICT; and techno-addiction, defined as a compulsive need for continuous interaction with technology (La Torre et al., 2020). These subtypes not only affect mental health but also the social and emotional lives of students, regardless of their age (Coppari et al., 2017). Although technostress generates adverse consequences, its proper management can lead to better emotional management and more efficient use of ICT, which can result in a more enriching educational experience.

A study conducted with Peruvian university students revealed that 49.4% of the participants experienced moderate levels of technostress, which negatively impacted their academic productivity and mental health (Ponce Pardo et al., 2023). The effects of technostress are more pronounced in women compared to men (Asensio-Martínez et al., 2023). Constant use of ICT contributes to a slight increase in technostress, affecting both the social and emotional lives of students. These effects are particularly concerning, as students’ psychological well-being directly influences their academic performance. However, when managed properly, the effects of technostress can present growth and personal development opportunities. Higher education teachers who face technostress critically and proactively turn this challenge into an opportunity to improve their digital skills and pedagogical practices (Nascimento et al., 2024). Additionally, improved emotional management helps teachers become more empathetic and effective in using ICT in their educational work (Castellanos-Alvarenga et al., 2024).

The advancement of ICT use, combined with the effects of technostress, underscores the importance of having validated tools to measure and address this phenomenon in the university population. The validation of psychometric instruments in different cultural contexts is essential to ensure their applicability and relevance. In this regard, Wang and Li (2019) developed a scale to measure technostress in higher education teachers in China, consisting of 22 items distributed across five factors: abilities-demand organizational misfit (ADO), needs-resources organizational misfit (NSO), abilities-technology demands misfit (ADT), needs-technology resources misfit (NST), and person–person misfit (PPF). Applied to a sample of 343 teachers, the scale showed a solid factorial structure and high internal consistency, supporting its validity in the academic context.

Penado Abilleira et al. (2020) adapted the scale into Spanish for a sample of 1,744 university students in Spain. Through exploratory factor analysis (EFA) and confirmatory factor analysis (CFA), the scale was adjusted to 20 items, confirming its high reliability and validity in the Spanish university context. More recently, Vega-Muñoz et al. (2022) adapted the instrument in Latin America with 212 students from the central-southern region of Chile to assess technostress in university students. The exploratory and confirmatory factor analysis showed excellent internal consistency, with a Cronbach’s alpha of 92.5% for the 19 remaining items, validating the scale for the Latin American context.

Given the above, it is essential to have validated tools to measure technostress in the university population. The aim of this study is to validate the 19-item version of the TS4US questionnaire in a sample of Peruvian students, addressing three key factors: abilities-technology-demands, needs-resources-supplies, and the person–person factor. This research is based on previous studies conducted by Wang and Li (2019) in China, Penado Abilleira et al. (2020) in Spain, and Vega-Muñoz et al. (2022) in Chile, whose results provide a solid foundation for this validation process in the Peruvian context.

## 2 Materials and methods

### 2.1 Design

Confirmatory Factor Analysis (CFA) is a fundamental statistical technique used to verify the structure of a predefined theoretical model by assessing whether the empirical data adequately fit the proposed factors. This technique is particularly useful in the validation of measurement instruments, especially those that use ordinal variables and Likert-type scales, which often exhibit skewness in the distribution of responses (Morata-Ramírez et al., 2015). CFA not only allows for the confirmation of construct validity but also the internal reliability of the questionnaire, employing various fit indices such as Chi-square, RMSEA, and other relevant indicators to evaluate the quality of the proposed model (Herrero, 2010).

### 2.2 Participants

The sample consisted of 814 students from various universities in Peru, of both sexes: women (451 = 55%) and men (363 = 45%), with ages ranging from 18 to 36 years (mean = 25.2 years; SD = 5.12). Most participants studied at private universities (83%) and were enrolled in face-to-face programs (80%). Geographically, 37% were from the coastal region, 34% from the highlands, and 26% from the jungle. In terms of faculties, 28% belonged to Health Sciences, 26% to Business Sciences, and 22% to Engineering and Architecture (Table 1).

**Table 1:** Sociodemographic information.

Category	Frequency	%
Age		
18–25 years	555	68%
26–35 years	164	20%
36 and older	95	12%
Gender		
Female	451	55%
Male	363	45%
Faculty		
Faculty of Health Sciences	227	28%
Faculty of Business Sciences	208	26%
Faculty of Humanities and Education	112	14%
Faculty of Engineering and Architecture	176	22%
Faculty of Theology	25	3%
Other programs	66	8%
Type of University		
Private	679	83%
Public	135	17%
Region of origin		
Coastal	300	37%
Highlands	276	34%
Jungle	210	26%
Foreign	28	3%
Study modality		
In-person	649	80%
Hybrid	165	20%

### 2.3 Instruments

The TS4US questionnaire (Technostress in University Students), initially developed by Wang and Li (2019) for teachers in China, has been adapted and validated for university students in Spain by Penado Abilleira et al. (2020). It measures the impact of excessive or inappropriate use of Information and Communication Technologies (ICT) on students’ well-being and academic productivity. For this study, the Latin American adaptation by Vega-Muñoz et al. (2022) was used. The questionnaire consists of 19 items distributed across three factors: Technoeducational Capabilities-Demands, Needs-Supplies Resources, and Person–Person Factor, using a 5-point Likert scale: Strongly Disagree = 1, Disagree = 2, Neither Agree nor Disagree = 3, Agree = 4, and Strongly Agree = 5. The TS4US has demonstrated a solid factorial structure and high internal consistency. Exploratory and confirmatory factor analyses revealed a three-factor structure with high validity and reliability. The confirmatory analysis showed good fit indices: RMSEA = 0.06, AGFI = 0.90, GFI = 0.92, and CFI = 0.95, while Cronbach’s alpha was 92.5%, supporting its reliability.

### 2.4 Data collection procedures

The study was conducted in several stages. First, a cultural adaptation was carried out through semi-structured interviews with eight higher education experts to assess the understanding of the items. Second, the adapted scale was evaluated by another eight specialist judges, who assessed the clarity, representativeness, and relevance of the items using a format that allowed for the calculation of Aiken’s V coefficient, thus determining content validity. Finally, a Google Forms questionnaire was used for data collection, which was active from March to July 2023. Participants were contacted via WhatsApp and institutional emails. Emphasis was placed on the importance of informed consent, ensuring that participation was completely voluntary.

### 2.5 Statistical procedures

The data analysis was structured in three phases. In the first phase, a preliminary analysis of the items by factors of the scale was conducted, evaluating the coefficients of skewness and kurtosis to determine the adequacy of the data distribution. In the second phase, a confirmatory factor analysis (CFA) was performed, analyzing the comparative fit index (CFI) and the Tucker-Lewis index (TLI), in addition to considering the parameters for the root mean square error of approximation (RMSEA) and the standardized root mean square residual (RMR), following the criteria proposed by Keith (2019). Finally, in the third stage, the reliability of the construct was determined using Cronbach’s alpha coefficient, yielding results that reflect high internal consistency. All analyses were carried out using SPSS Amos version 25 statistical software.

## 3 Results

Table 2 shows the preliminary analysis of the 22 items derived from the original model, where it is observed that item 7 has the highest mean (*M* = 2.66), while items 9 and 10 show greater variability (SD = 1.13). Additionally, it is observed that the skewness and kurtosis are within the ±1.5 range, indicating a normal distribution (Pérez and Medrano, 2010).

**Table 2:** Descriptive analysis of the items.

Items	*M*	SD	g1	g2
TE1	2.57	1.07	0.3	−0.58
TE2	2.55	1.07	0.37	−0.65
TE3	2.46	1.12	0.5	−0.56
TE4	2.41	1.11	0.55	−0.47
TE5	2.58	1.1	0.3	−0.72
TE6	2.63	1.12	0.26	−0.77
TE7	2.66	1.11	0.23	−0.71
TE8	2.65	1.12	0.31	−0.67
TE9	2.54	1.13	0.38	−0.66
TE10	2.59	1.13	0.36	−0.68
TE11	2.52	1.11	0.4	−0.6
TE12	2.39	1.06	0.48	−0.43
TE13	2.48	1.09	0.41	−0.65
TE14	2.53	1.1	0.37	−0.64
TE15	2.38	1.07	0.51	−0.41
TE16	2.47	1.08	0.39	−0.53
TE17	2.44	1.07	0.4	−0.6
TE18	2.37	1.08	0.61	−0.23
TE19	2.44	1.05	0.47	−0.39
TE20	2.47	1.06	0.4	−0.51
TE21	2.48	1.09	0.48	−0.47
TE22	2.6	1.12	0.33	−0.65

*M = mean, SD = standard deviation, g1 = skewness, g2 = kurtosis.*

### 3.1 Exploratory factor analysis

An Exploratory Factor Analysis (EFA) was conducted, considering the versions proposed by Wang and Li (2019) with 22 items (Model 1), Penado Abilleira et al. (2020) with 20 items (Model 2), Vega-Muñoz et al. (2022) with 19 items (Model 3), and a new version with 11 items for Peruvian university students (Model 4) (Table 3).

**Table 3:** Exploratory factor analysis of the different versions of the technostress scale.

Model 3. EFA of the 22-item model by Wang and Li (2019)				
	F1	F2	F3	F4
Items				
TE20	0.9			
TE21	0.85			
TE19	0.85			
TE22	0.83			
TE18	0.64			
TE17	0.57			
TE16	0.47			0.38
TE8		0.9		
TE9		0.87		
TE7		0.85		
TE6		0.8		
TE10		0.55		
TE11		0.41	0.38	
TE4			0.88	
TE3			0.88	
TE2			0.81	
TE1			0.8	
TE5			0.57	
TE13				0.64
TE12				0.63
TE14		0.33		0.46
TE15	0.31			0.45
% Variance	0.24	0.21	0.2	0.12
% Cumulative variance	0.24	0.45	0.65	0.78
Factor correlation				
Factors				
F1	1			
F2	0.68	1		
F3	0.64	0.73	1	
F4	0.68	0.5	0.61	1

Model 4. EFA of the 20-item model by Penado Abilleira et al. (2020)				
	F1	F2	F3	F4
Items				
TE20	0.92			
TE19	0.89			
TE21	0.89			
TE22	0.86			
TE18	0.74			
TE17	0.69			
TE16	0.62			
TE8		0.93		
TE7		0.89		
TE9		0.88		
TE6		0.84		
TE10		0.66		
TE11		0.52		
TE3			0.89	
TE4			0.89	
TE2			0.85	
TE1			0.82	
TE5			0.55	
TE12	0.36			0.55
TE13	0.44			0.48
% Variance	0.29	0.23	0.2	0.06
% Cumulative variance	0.29	0.51	0.72	0.78
Factor correlation				
Factors				
F1	1			
F2	0.71	1		
F3	0.67	0.76	1	
F4	0.49	0.34	0.52	1

Model 5. EFA of the 19-item model by Vega-Muñoz et al. (2022)				
	F1	F2	F3	F4
Items				
TE19	0.88			
TE20	0.86			
TE21	0.83			
TE18	0.68			
TE17	0.61			
TE16	0.49			0.37
TE8		0.88		
TE7		0.84		
TE9		0.84		
TE6		0.8		
TE3			0.88	
TE4			0.88	
TE1			0.8	
TE2			0.79	
TE11		0.37	0.39	
TE13				0.66
TE12				0.65
TE14		0.33		0.51
TE15				0.49
% Variance	0.24	0.21	0.2	0.14
% Cumulative variance	0.24	0.45	0.65	0.79
Factor correlation				
Factors				
F1	1			
F2	0.68	1		
F3	0.64	0.71	1	
F4	0.72	0.52	0.62	1

Model 6. EFA of 11 items in Peruvian university students			
	F1	F2	F3
Items			
TE8	0.93		
TE7	0.9		
TE6	0.88		
TE9	0.83		
TE20		0.92	
TE21		0.89	
TE19		0.88	
TE22		0.87	
TE1			0.95
TE2			0.89
TE3			0.65
% Variance	0.29	0.29	0.2
% Cumulative variance	0.29	0.59	0.79
Factor correlation			
Factors			
F1	1	0.72	0.74
F2	0.72	1	0.65
F3	0.74	0.65	1

Before performing the factor analysis, a parallel analysis was conducted to identify how many factors were present, considering the number of items in each version. In the versions by Penado Abilleira et al. (2020), Vega-Muñoz et al. (2022), and Wang and Li (2019), 4 factors were found. The Kaiser-Meyer-Olkin (KMO) index for the three versions showed an optimal level (0.97, 0.96, 0.96, respectively), and the Bartlett’s Test of Sphericity was statistically significant for all three versions (*p* < 0.001). On the other hand, the 11-item version for Peruvian university students showed the presence of 3 factors, with a KMO of 0.93 and a significant Bartlett’s Test of Sphericity (*p* < 0.001).

Although the factor loadings for all versions were above 0.3, in Model 1, the lowest factor loading was 0.41 for item 11, while items 20 and 8 had the highest factor loadings (0.90), and the correlations between factors were above 0.50, with an accumulated variance of 78%. Likewise, in Model 2, item 13 had the lowest factor loading (0.48), and item 8 had the highest factor loading (0.93), with an accumulated variance of 78%, and the correlations between factors were above 0.34. Model 3 shows that item 14 had the lowest factor loading (0.33), and the items with the highest factor loadings were items 19, 8, and 3 (0.88). The factor loadings’ correlations were above 0.52, with an accumulated variance of 79%. Finally, in the version for Peruvian university students (Model 4), item 3 had the lowest loading, while item 1 had the highest factor loading. Additionally, the correlation between factors was above 0.65, and the accumulated variance reached 79%.

**Figure 1:**
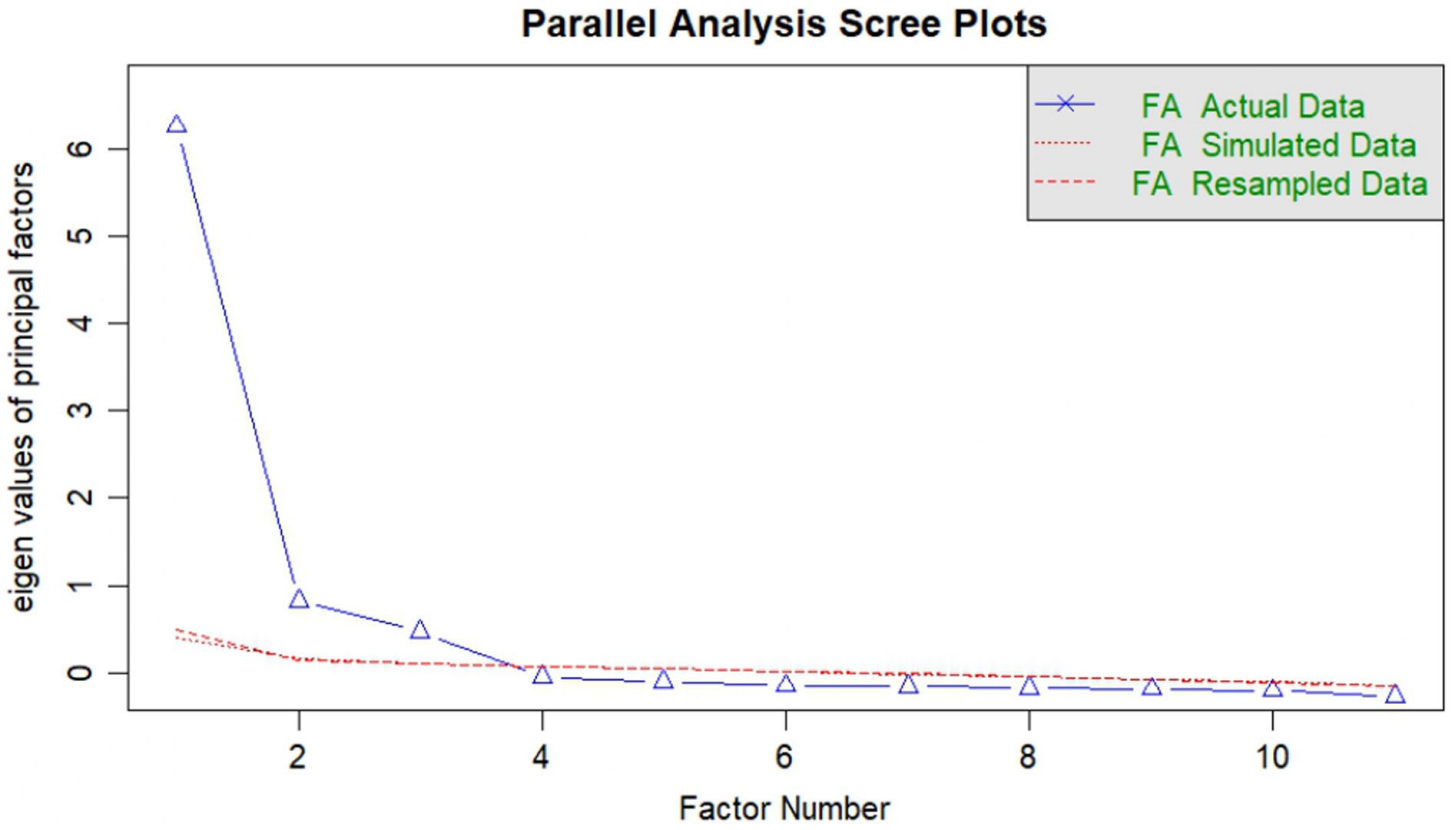
Parallel analysis of the version for Peruvian university students (11 items).

It is important to clarify that the original model reports the presence of 3 dimensions. After eliminating the items according to each version, none showed an adequate fit, as the first three versions revealed the presence of 4 factors. Moreover, the items were grouped into dimensions that they do not theoretically belong to. Therefore, a version with 11 items was proposed, maintaining the distribution of the 3 original dimensions (Figure 1), achieving a balance between the theoretical and numerical aspects.

### 3.2 Confirmatory factor analysis and invariance by gender

**Figure 2:**
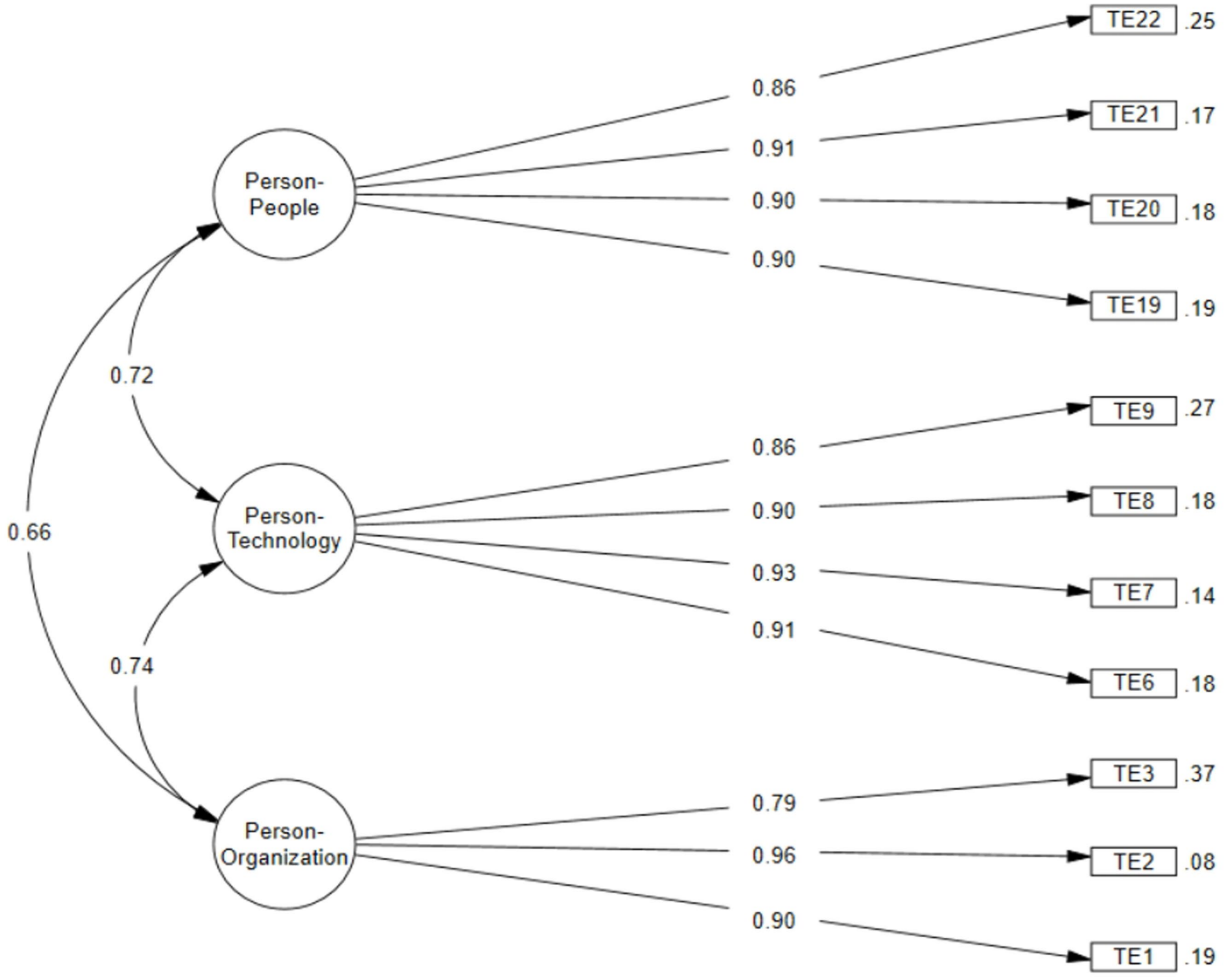
Internal structure of the tecnostress scale for Peruvian university students.

A Confirmatory Factor Analysis (CFA) was conducted for each version proposed earlier (Table 4). First, it is observed that the version by Wang and Li (2019) proposed 5 dimensions with 22 items (Model 1), but it did not provide a good fit for the Peruvian population. The same occurred with the version by Penado Abilleira et al. (2020), which also proposed 5 dimensions, but with 20 items (Model 2). This version achieved an adequate CFI but did not show good results in the TLI or RMSEA. Similarly, the version proposed by Vega-Muñoz et al. (2022), which included 3 dimensions and 19 items (Model 3), also did not achieve an adequate fit. Therefore, a new version with 11 items grouped into 3 dimensions (Model 4) was created, as proposed in the EFA, maintaining the independence of each item within the theoretical factor proposed. This new version showed an adequate fit with a CFI = 0.982, TLI = 0.975, RMSEA = 0.007, and SRMR = 0.02, where the factor loadings for each item were above 0.86, and the correlations between the dimensions were above 0.66 (Figure 2).

**Table 4:** Fit indices of the confirmatory factor analysis of the different versions of the tecnostress scale for Peruvian university students.

Models	p-X2	Df	CFI	TLI	RMSEA	SRMR
Model 1	0.001	179	0.894	0.876	0.122	0.045
(5 dimensions – 22 items)						
Model 2	0.001	190	0.908	0.891	0.116	0.043
(5 dimensions – 20 items)						
Model 3	0.001	136	0.817	0.785	0.178	0.068
(3 dimensions – 19 items)						
**Model 4**	**0.001**	**41**	**0.982**	**0.975**	**0.07**	**0.02**
**(3 dimensions – 11 items)**						

Df = Degrees of Freedom, CFI = Comparative Fit Index, TLI = Tucker-Lewis Index RMSEA = Root Mean Square Residual, RMSR: Root Mean Square Residual. Note: Model 4. (3 dimensions – 11 items) = New version of the technostress scale for students.

On the other hand, Table 5 shows the invariance by gender, where it was observed that the difference in CFI (ΔCFI) between configural-metric, metric-scalar, and scalar-strict models was less than 0.010, and the difference in RMSEA (ΔRMSEA) was less than 0.015. This indicates that the 3-dimensional model with 11 items presents measurement invariance for both men and women, suggesting that this version is suitable for measuring Technostress in both genders without distinction.

**Table 5:** Factorial invariance analysis by gender (Males = 363 and Females = 451).

Model	χ^2^	Δχ^2^	gl	Δgl	*p*	CFI	ΔCFI	RMSEA	ΔRMSEA
Configural	17.48934		82		0.001	0.994		0.011	
Metric	38.97925	−21.48991	90	−8	0.001	0.983	0.011	0.022	−0.011
Scalar	42.24986	−3.27061	98	−8	0.001	0.982	0.001	0.022	0.001
Strict	47.88721	−5.63735	109	−11	0.001	0.981	0.001	0.022	0.001

Δχ^2^, Variation in the chi-square test; Δdf, Variation in degrees of freedom; *p*, probability; ΔCFI, Variation in the comparative fit index; ΔRMSEA, Variation in the root mean square error of approximation.

### 3.3 Internal consistency

The internal consistency of the items showed adequate values in both the ordinal Alpha coefficient and Omega, where it was observed that the Person–Person dimension had an *α* = 0.91 and an *ω* = 0.91. Regarding the Person-Technology dimension, it presented an *α* = 0.92 and an *ω* = 0.92. Lastly, the Person-Organization dimension obtained an *α* = 0.86 and an *ω* = 0.98, which indicates that the technostress scale exhibits good internal consistency.

## 4 Discussion

This study validated the TS4US instrument in the context of higher education in Peru, making a comparison with the results of previous research conducted in Spain, Chile, and China. The sample consisted of Peruvian university students, whose characteristics are similar to those of the populations studied in those countries, facilitating a valid comparison of the results.

The final TS4US model in Peru, with 11 items and 3 dimensions, demonstrated strong fit indices: CFI = 0.982, TLI = 0.975, and RMSEA = 0.07. These values surpassed those of more complex versions, such as the 19-item version (CFI = 0.817, TLI = 0.785, and RMSEA = 0.178). Despite a solid theoretical foundation, the Vega-Muñoz et al. (2022) model did not capture the interrelationships between the dimensions of techno-stress as precisely. In contrast, the 11-item version optimizes both the structure and applicability, making it a more efficient tool for assessing techno-stress in the Peruvian context.

The adoption of a three-factor structure in Peru and Chile reflects a trend toward model simplification without sacrificing effectiveness. This allows the questionnaire to remain relevant in educational contexts with high exposure to ICTs, optimizing its practical utility. In Peru, the model achieved outstanding internal consistency, with a Cronbach’s alpha of 0.964 for the total scale, and all individual factors exceeded 0.90, demonstrating its robustness.

The Chilean model also showed strong performance, with a Cronbach’s alpha of 0.925 for the 19-item version, validating its use in the Latin American context. Although previous studies, such as those by Penado Abilleira et al. (2020) and Wang and Li (2019), employed more complex models with alphas ranging from 0.91 to 0.95, the results obtained with the simplified TS4US version for Peru and Chile highlight the instrument’s versatility, adapting to various cultural and educational contexts.

This study has significant implications for higher education institutions. The validation of TS4US in the Peruvian context provides a useful tool for monitoring and evaluating techno-stress in students, enabling universities to identify problem areas related to ICT use. By better understanding the dimensions of techno-stress, such as the mismatch between students’ skills and technological demands or the lack of organizational resources, universities can design effective interventions. These could include training teachers in ICT use, strengthening social support for students, or improving technological infrastructure. Moreover, adjusting the model to the cultural and contextual characteristics of Peruvian students could enhance the effectiveness of strategies to reduce techno-stress, promoting student well-being and academic performance.

Although the findings are valuable, there are some limitations. As a cross-sectional study, it is not possible to observe how techno-stress levels change over time. Longitudinal studies would be helpful to assess these changes. It would also be advisable to include variables such as social support and coping strategies, which may influence techno-stress levels and provide a more comprehensive view of the phenomenon. Expanding the sample and considering other geographic and cultural contexts could improve the generalization of the results and help validate the models in a broader range of situations.

This study confirms the validity and reliability of the TS4US as a tool for measuring techno-stress in Peruvian university students. The results support its ability to adapt to different cultural and educational contexts, as evidenced by comparisons with previous studies in Chile, Spain, and China. The appropriate model fit in the Peruvian population emphasizes the importance of adapting instruments to the specific characteristics of each population to ensure accurate results.

The findings suggest that TS4US could be a valuable tool for universities to identify key factors related to techno-stress. This study provides a solid foundation for the creation of strategies that promote student well-being, highlighting the need to adjust educational policies to technological demands and provide additional support for students.

Finally, the results of this study open the door to future research and improvements in higher education. Implementing programs to support the proper use of ICTs and regularly evaluating the impact of technology on students can help create a more inclusive and resilient educational environment in the face of technological challenges.

## Data Availability

The original contributions presented in the study are included in the article/supplementary material, further inquiries can be directed to the corresponding author.
